# Novel Mutant Phospholipase D from *Hemiscorpius lepturus* Acts as A Highly Immunogen in BALB/c Mice Against the Lethality of Scorpion Venom

**DOI:** 10.3390/molecules25071673

**Published:** 2020-04-04

**Authors:** Abouzar Soleimani Moez, Reza H. Sajedi, Kamran Pooshang Bagheri, Jean-Marc Sabatier, Delavar Shahbazzadeh

**Affiliations:** 1 Department of Biochemistry, Faculty of Biological Sciences, Tarbiat Modares University, Tehran P.O. Box 14115-111, Iran; A_soleimani@modares.ac.ir; 2 Venom and Biotherapeutics Molecules Lab., Medical Biotechnology Department, Biotechnology Research Center, Pasteur Institute of Iran, Tehran P.O. Box 13169-43351, Iran; k_bagheri@pasteur.ac.ir; 3 Institute of Neuro Physiopathology (INP), Université d’Aix-Marseille, UMR 7051, Faculté de Pharmacie, 27 Bd Jean Moulin, 13385 Marseille Cedex, France; sabatier.jm1@libertysurf.fr

**Keywords:** *Hemiscorpius lepturus (H. lepturus)*, phospholipase D (PLD), Freund’s adjuvant, alum adjuvant, dermonecrosis, vaccine, Splenocytes proliferation assay

## Abstract

*Hemiscorpius lepturus (H. lepturus)* which belongs to the Scorpionidae family, is the deadliest scorpion in Iran. It causes pathological manifestations like dermonecrosis, hemolysis, renal failure, necrotic ulcers, and in some cases, even death. The venom of this scorpion is well-known for its cytotoxic effects in comparison with the other venomous scorpions which show significant neurotoxic effects. Due to the painless nature of the sting of this scorpion, the clinical symptoms occur in victims 24 to 72 h post-sting. In our previous studies during the last decade, we demonstrated that the medical complications are attributable to the presence of phospholipase D (PLD) as a major toxin in the venom. With the purpose of designing and constructing a vaccine against *H. lepturus* for humans, animal model experiments were performed. To achieve this goal, non-toxic PLD was developed by mutation of two critical catalytic residues—His12 and His48—into alanines and the product was then denominated mut-rPLD1. The in-vivo tests showed that the mice immunized with interval doses of 10 µg of mut-rPLD1, were completely protected against 10× the LD_100_ of the venom. In conclusion, this mutant may be an effective vaccine candidate against scorpion envenomation by *H. lepturus* in future clinical studies.

## 1. Introduction

The envenomation by *H. lepturus* is one of the greatest problems from a clinical viewpoint in the southwestern area of Iran, especially in Khuzestan Province [1]. This phenomenon has been recurrently reported in Iran [2]. This scorpion belongs to the Hemiscorpiidae family [3], while other medically important scorpions such as *Odontobuthus doriae*, *Mesobuthus eupeus*, *Androctonus crassicauda*, *Buthotus saulcyi* and *Buthotus sach* that are responsible for scorpionism, belong to the large family Buthidae [4,5]. Although *H. lepturus* is only responsible for about 15% of scorpion stings throughout Iran, but due to the nature of its venom it accounts for about 95% of all mortalities caused by scorpion stings [6]. The venom of *H. lepturus* has several characterized peptides and enzymes such as hemicalcin, hemitoxin, hemilipin and heminecrolysin (HNC) which trigger erythrocyte lysis, anti-microbial peptides (AMPs), phospholipases such as A_1_, A_2_, B, C and D [3,7,8]. According to the painless nature of its venom and slow development of symptoms in the victims, the stung patients mostly fail to go to health centers to get medical treatment [9]. The most rigorous toxicity and lethality has been mainly observed in children [10]. Some of its complicated symptoms include nephrotic syndrome, renal failure, dermonecrotic lesions, erythema and immediate death [11,12]. Other clinical manifestations include persistent inflammation, vascular leakage, cardiovascular diseases, platelet aggregation, severe hemolysis and hematuria, which ultimately lead to renal failure [6,9,13,14]. Hepatic injuries were also observed based on increased leakage of some key hepatic enzymes such as glutamate-oxaloacetate transaminase (SGOT), glutamate-pyruvate transaminase (SGPT) and alkaline phosphatase (ALKP) which are all related to the potential after-effects of *H. lepturus*-venom [15]. The main clinical symptoms of *H. lepturus* venom are very similar to those caused by the brown spider *Loxosceles intermedia* [16]. The main toxicity and lethality properties of *H. lepturus*-venom are attributed to phospholipase D1 (PLD1) activity [17,18]. It has been proven by ClustalW analysis that most of the PLDs from the *Loxoscelidae* family have high structural and functional homology with *H. lepturus*-PLD1 (a 32 KDa protein) [19]. In the previous studies, all of these phospholipases were characterized as sphingomyelinase D (SMase D) which hydrolyzes sphingomyelin (SM) in the outer leaflet of plasma membrane and neuron myelin sheath into ceramide-1-phosphate and choline [20,21,22,23]. These enzymes also have PLD activity; in which they hydrolyze lysophosphatidylcholine (LPC) into lysophosphatidic acid (LPA) and choline. Some of these metabolites are significant lipid mediators which in turn cause many pathological and physiological developments [24,25,26]. In 2013, Lajoie et al. by using ^31^P-NMR and mass spectrometry demonstrated that recombinant PLD of *Loxosceles* species doesn’t hydrolyze LPC to LPA and choline via a simple hydrolysis reaction or hydrolyze sphingomyelin to ceramide-1-phosphate and choline, but rather this enzyme exclusively catalyzes a rare transphosphatidylation reaction forming cyclic phosphate metabolites from both these main substrates. In this regard PLD converts 1-palmitoyl-2-hydroxy-*sn*-glycero-3-phosphocholine (palmitoyl LPC) to 1-palmitoyl-*sn*-glycero-2,3-cyclic-phosphatidic acid (palmitoyl CPA) and converts 6:0 SM N-hexanoyl-D-*erythro*-sphingosylphosphorylcholine (SM) into cyclic ceramide-(1,3)-phosphate (CC(1,3)P) [27]. Hence according to these findings, *H. lepturus*-PLD1 most probably causes the same enzymatic reactions. These poorly soluble cyclic phosphates may be relevant to the pathology of envenomation which ultimately results in death. Since the PLD is mainly responsible for toxicity and lethality in humans, it is necessary to develop a new recombinant non-toxic PLD vaccine for preventing the complications caused by *H. lepturus* scorpion envenomation. In this study we designed and constructed a new non-toxic mutant of *H. lepturus*-PLD1 in which its two critical catalytic residues—His12 and His48—were substituted with Ala and thus recombinant PLD1 was denominated mut-rPLD1 (His12Ala—His48Ala) was developed. In this study, the potency of this molecule to act as a vaccine in order to neutralize the lethality of whole venom of *H. lepturus* scorpion was evaluated in a mice model.

## 2. Results

### 2.1. Protein Structure Analysis 

**Figure 1:**
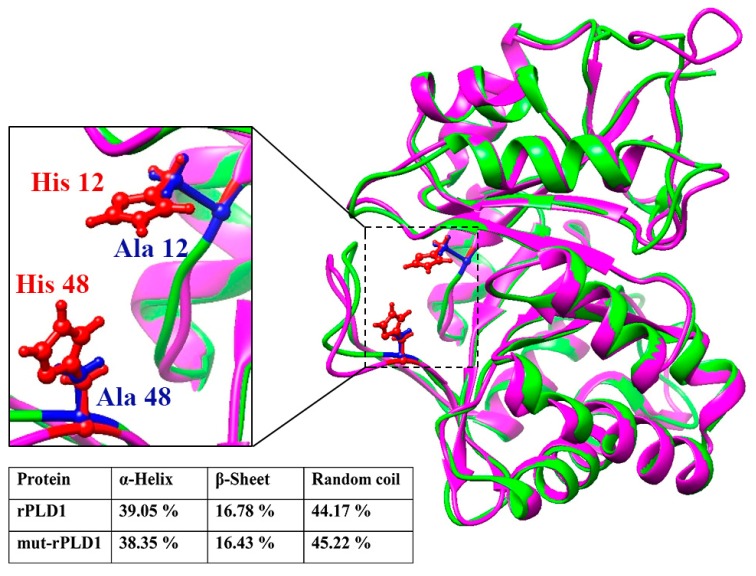
Superimposition of the structure of rPLD1 with the structure of mut-rPLD1. The backbone of rPLD1 is presented as pink and for mut-rPLD1 as green. Both structures have TIM β/α Barrel fold and show evidence of high structural homology. In the magnified close-up view (left) the His12 and His48 of rPLD1 are shown in red and Ala12 and Ala48 of mut-rPLD1 in blue. The percentages of the secondary structures are depicted in inserted table.

The RMSD (Root Mean Square Deviation) value for rPLD1 was estimated to 5.7 ± 3.6 Å and for mut-rPLD1 was 6.0 ± 3.7 Å. Accordingly, a difference of 0.3 Å in RMSD indicates extreme structural homology between the two intended proteins. Finally, structural superimposition of rPLD1 with mut-rPLD1 was performed by using the UCSF Chimera software (Figure 1). In accordance with the I-TASSER server’s calculations, of the 292 rPLD1-amino acid residues, 114 of them are in the α-helix, 49 of them in the β-sheet and 129 in the random coil configurations. Consequently, α-helixes, β-sheets and random coils make up 39.05%, 16.78% and 44.17% respectively of the rPLD1 structure. As for the mut-rPLD1, 112 residues are in the α-helix, 48 in the β-sheet and 132 in the random coil configurations, which in turn make up 38.35%, 16.43% and 45.22% respectively of the mut-rPLD1 structure (Figure 1). Using the AntheProt 3D software, extremely close results were also obtained (data not shown).

### 2.2. Circular Dichroism

**Figure 2:**
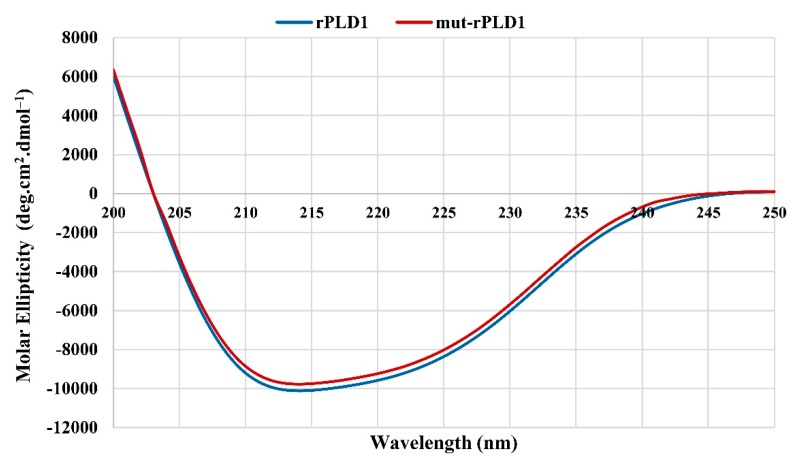
Circular dichroism spectra for the rPLD1 and mut-rPLD1. Spectra were collected using proteins in 1X PBS, pH 7.4, at 25 °C. Molar Ellipticity was studied in a wavelength range from 190 to 250 nm.

Circular dichroism analysis displayed similar spectra for both rPLD1 and mut-rPLD1, proving that the mutation of His12 and His48 to Ala did not change the overall secondary structure of rPLD1. As can be viewed in Figure 2, the secondary structure spectrum for the mut-rPLD1 is extremely similar to the rPLD1 circular dichroism profile.

### 2.3. Fluorescence Spectroscopy

**Figure 3:**
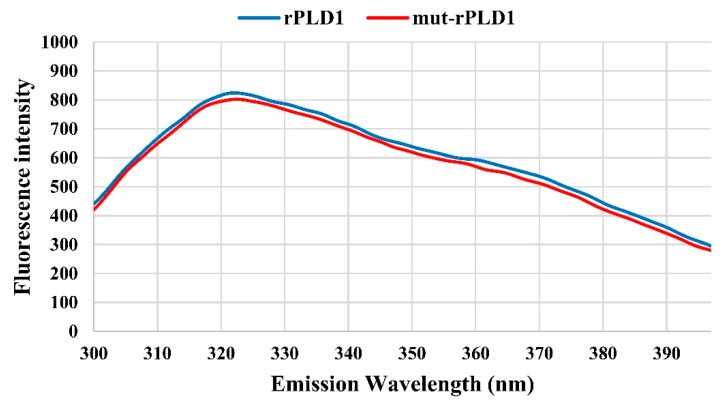
Intrinsic fluorescence emission of rPLD1 and mut-rPLD1. As seen here, both proteins have the similar fluorescence emission pattern. λ_ex_ = 280 nm with excitation slit of 10 nm, λ_em_ = 300 to 400 nm with emission slit of 5 nm. Both proteins have maximum emission at wavelength equal to 322 nm.

The tryptophan fluorescence emission spectra of both rPLD1 and mut-rPLD1 follow a common pattern. As depicted in Figure 3, both proteins have the highest emission at a wavelength of 322 nm. No red or blue shifts were observed with respect to their fluorescence emission. These data strongly indicate that the mutation of His12 and His48 to Ala does not induce changes in the three-dimensional structure of the mut-rPLD1 and thus both this compound and rPLD1 have the same three-dimensional structure and identical epitopes, which in turn identifies mut-rPLD1 as a non-toxic alternative to rPLD1.

### 2.4. SDS-PAGE

**Figure 4:**
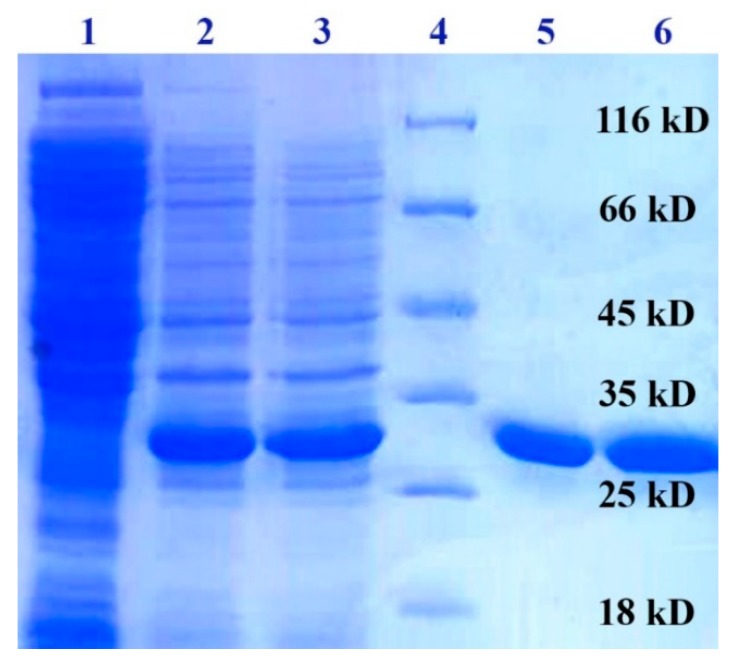
SDS-PAGE analysis of expressed and purified rPLD1 and mut-rPLD1. Lane 1 shows uninduced *E. coli* BL21, lane 2, induced bacteria containing rPLD1, lane 3, induced bacteria containing mut-rPLD1, lane 4, protein size marker (Thermo Fisher Scientific Co. Waltham, MA, USA), lane 5, purified rPLD1 and lane 6, purified mut-rPLD1. Both proteins settled at the expected weight of 32 KDa.

The mut-rPLD1 showed a single band fully identical to rPLD1 with the expected molecular weight at 32 KDa (Figure 4).

### 2.5. TNPAL-SM Hydrolysis

**Figure 5:**
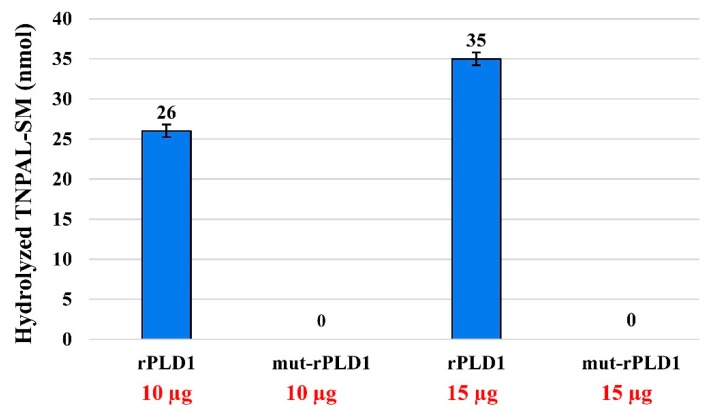
Sphingomyelinase activity of rPLD1 and mut-rPLD1. The substrate TNPAL-SM was incubated with different amounts (10 and 15 µg) of both rPLD1 and mut-rPLD1. As illustrated here, the quantity of the rPLD1 activity is dose-dependent but mut-rPLD1 lacks any enzymatic activity in any dose. 1 nmol of hydrolyzed TNPAL-SM corresponds to 0.023 absorbance units.

In regard of the biochemical outcomes, rPLD1 in amounts of 10 and 15 µg was able to hydrolyze 26 and 35 nmol of TNPAL-SM, respectively. However, at these same amounts, mut-rPLD1 was not able to hydrolyze any dosages of TNPAL-SM (Figure 5). These results strongly demonstrate that the mutation of His12 and His48 to Ala has led to complete inactivation of the enzyme.

### 2.6. Non-Lethality Assay of Mut-rPLD1

All four groups of mice that received 3.7, 18.5, 37 and 111 µg of mut-rPLD1 respectively, survived 2 weeks after intraperitoneal injection, while the mice in the negative control group that received 3.7 µg of rPLD1 were dead during the 12 h post-injection. This survival rate affirmed our hypothesis that mutation of His12 and His48 to Ala resulted in complete inactivation of the enzyme (Table 1).

### 2.7. Non-Dermonecrotic Test of Mut-rPLD1

**Figure 6:**
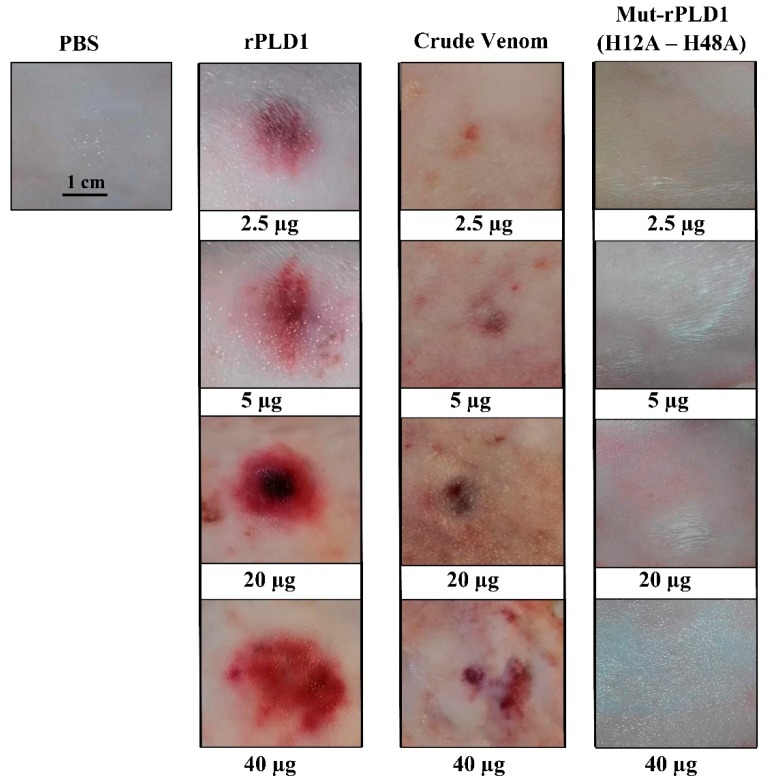
Dermonecrotic test in rabbit skin. Macroscopic visualization of skin lesions by intradermal injection of 2.5, 5, 20 and 40 µg of rPLD1, crude venom and mut-rPLD1 at 24 h post-injection. As displayed here, both rPLD1 and CV affect clear dermonecrotic lesions, however mut-rPLD1 has no dermonecrotic effect on rabbit skin. Phosphate buffered saline (PBS) was injected as negative control.

There were no necrotic lesions due to any of the four intradermal injected doses of mut-rPLD1 in rabbit skin at 6, 12, 24, 48 and 72 h post-injection. This phenomenon strongly confirms that the mutation of His12 and His48 to Ala has led to total inactivation of the enzyme. Necrotic areas and inflammatory reactions attributed to all doses of rPLD1 could be assessed by the presence of erythema, edema and mild swelling within about 12 h after injection. Also these necrotic areas and inflammatory reactions due to all tested amounts of crude venom was observed over the same time interval (Figure 6).

### 2.8. Vaccination Trials in the Mice

The results indicated that the mice vaccinated with mut-rPLD1-Freund/Alum adjuvants challenged either by rPLD1 or crude venom of *H. lepturus*, survived throughout the study. The results obviously indicated that all mice vaccinated with the mut-rPLD1 were completely protected against a wide range of rPLD1 or crude venom concentrations, using either of these two adjuvants [Table 2]. Non-immunized negative control mice in all groups died after injecting one LD_100_ of rPLD1 or one LD_100_ of crude venom. From these results, it can be deduced that the amount of vaccine (mut-rPLD1 = 10 µg/mouse) which promoted the efficacy of the immune response was certainly adequate to protect the mice against rPLD1 or crude venom of *H. Lepturus*.

### 2.9. ELISA Test

The results showed the considerable improvement in targeting all antigens as described below: the anti-toxoid sera from Freund-immunized mice interacted with rPLD1, mut-rPLD1 and crude venom.

**Figure 7:**
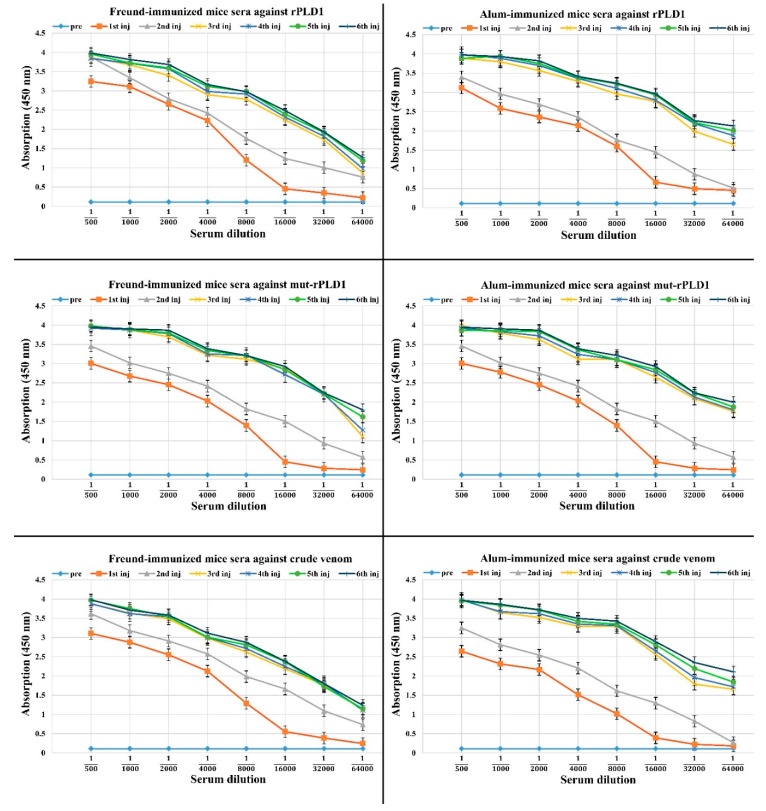
The output of ELISA when mice antisera executed against rPLD1, mut-rPLD1 and crude venom in both group of mice whether immunized by Freund or Alum adjuvants. (Pre) indicates pre-immunized serum. The titration profile reveals the triplicate assay ± SD.

In regard to Freund-immunized mice sera against rPLD1 test, a rising titer of antibody was observed after the first injection of mut-rPLD1 at 1:500 serum dilution and remained at high level at 1:8000. At the serum dilution of 1:8000, the serum reacts with rPLD1 at optical density (OD) = 1.23, with mut-rPLD1 at OD = 1.41 and with crude venom at OD = 1.37. Consequently, optimum titer was observed at approximately 1:8000 serum dilution at the first injection. The amount of anti-mut-rPLD1 serum peaked after the 3rd injection and from this point onwards; after the 4th, 5th and 6th injections, the antibody titers reached a plateau state. These titers indicate an effective and successful immunization in the mice. In the other five experiments all have similar and very close results as shown in Figure 7.

### 2.10. Ouchterlony Test

**Figure 8:**
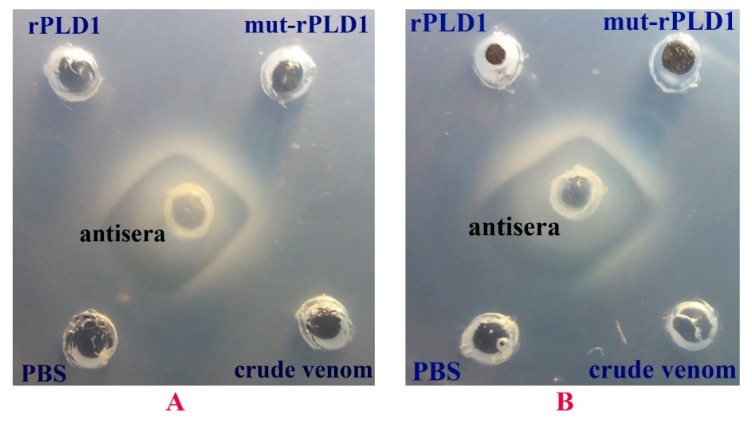
Ouchterlony tests. (**A**) Cross-Reactivity of IgG in the antisera of Freund’s-immunized mice with rPLD1, mut-rPLD1 and Crude venom led to formation of the precipitin lines without any spur that strongly indicates that there is a full identity between all three antigens. (**B**) The same cross-reactivity was applied for Alum-immunized mice. PBS was used as negative control.

The antibodies in the antisera and also all three other antigens diffused radially out of their respective holes into the gel. As illustrated in Figure 8 the antisera diffusion front met all three antigens; rPLD1, mut-rPLD1 and crude venom, recognized them and interacted and bound to all of them which led to formation of large aggregates of antigen-antibody complexes which precipitated in the gel that can be visualized as a white line so-called precipitin line. The formation of continuous arc line without formation of any spur strongly corroborates that antibody in the antisera is not only capable of binding to all of them but also this phenomenon shows full identity between rPLD1, mut-rPLD1 and native PLD in the whole crude venom of *H. lepturus* and therefore they all have the same antigenic epitopes.

### 2.11. Western Blotting

**Figure 9:**
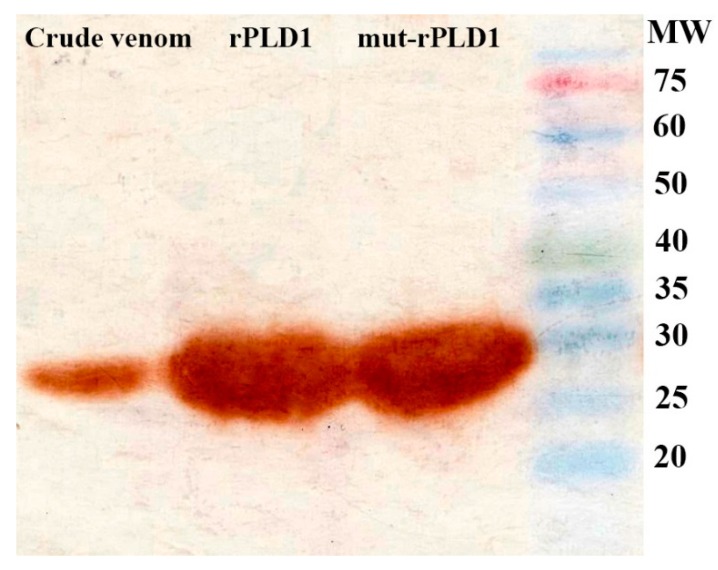
Immunoreactivity results of mice anti-mut-rPLD1 sera against crude venom, rPLD1 and mut-rPLD1 by Western blotting. All three samples were subjected to 15% SDS-PAGE, crude venom, rPLD1 and mut-rPLD1 were transferred to a PVDF membrane. The membrane was incubated with anti-sera (diluted at 1:1000 titer) followed by incubating with HRP-labelled Goat anti-mouse IgG (diluted at 1:3000 titer). Ultimately, all the relevant bands came into view.

The results showed immunoreactivity of mice anti-toxoid sera against rPLD1, mut-rPLD1 and crude venom, confirming that IgG molecules formed in the mice are able to bind Hl-PLD and neutralize its toxic and lethal effects (Figure 9).

### 2.12. Effect of Antigens on Splenocytes Proliferation

**Figure 10:**
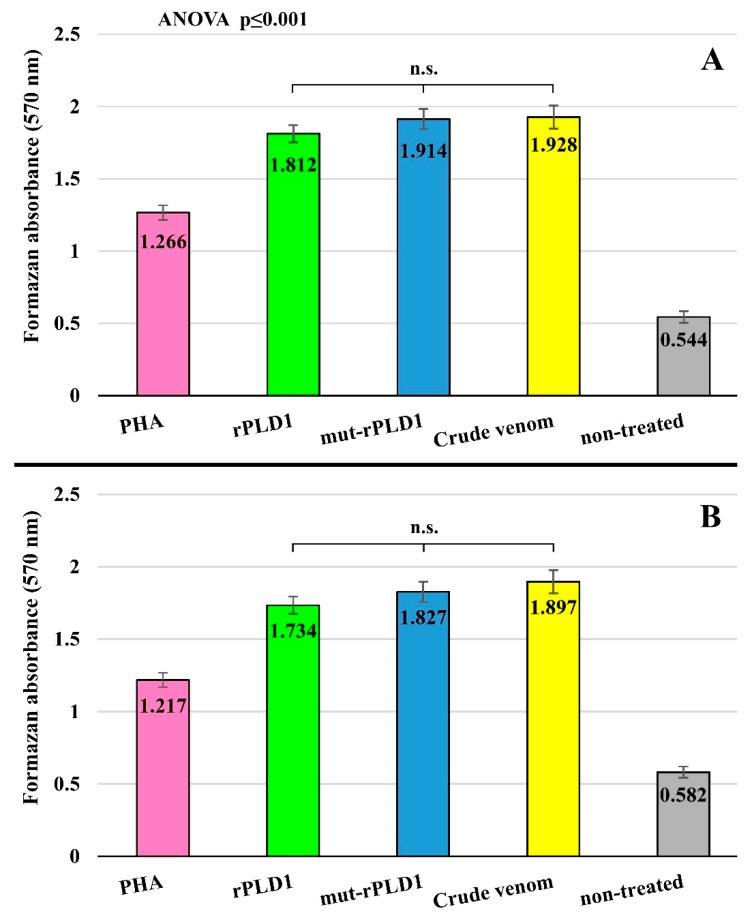
Conventional MTT assay was performed by absorption at 570 nm in regard to 4 groups of splenocytes treated with PHA, rPLD1, mut-rPLD1 and crude venom after 24 h post-treating. Non-treated group absorbance collection was done as negative group. (**A**) Assay was performed on Freund-immunized mice. (**B**) Assay was performed on Alum-immunized mice.

Regarding to Freundʹs mut-rPLD1-immunized mice, it was ascertained that rPLD1, mut-rPLD1 and crude venom significantly stimulated splenocyte proliferation compared to non-treated groups and even in comparison with phytohemagglutinin (PHA), so that in this respect the mean absorptions of formazan for PHA, rPLD1, mut-rPLD1, crude venom and non-treated groups were 1.266, 1.812, 1.914, 1.928 and 0.544, respectively (Figure 10A). As a result, the stimulation indexes for PHA, rPLD1, mut-rPLD1 and crude venom were 2.32, 3.33, 3.51 and 3.54, respectively. Concerning the alum mut-rPLD1-immunized mice, extremely close results were observed as expressed here. The mean absorptions of formazan for PHA, rPLD1, mut-rPLD1, crude venom and non-treated groups were 1.217, 1.734, 1.827, 1.897 and 0.582, respectively (Figure 10B). Thus the stimulation indexes for PHA, rPLD1, mut-rPLD1 and crude venom in this group were 2.09, 2.97, 3.13 and 3.25, respectively.

Hence by comparing the aforementioned results it is deduced that all three antigens can act as a potent mitogen whenever the spleen encounters them. Subsequently it can be acknowledged whenever the venom enters the body, the splenocytes turn into vast proliferative phase, by this means countering the toxic and lethal effects of the venom via producing immense level of antibody and trigger effective and suitable immune response.

## 3. Discussion

*H. lepturus* is one of the most dangerous scorpions in Iran from a clinical perspective especially in Khuzestan Province [28]. The venom of *H. lepturus* is highly toxic and contains hemotoxins and cytotoxins such as heminecrolysin [17]. In this regard, PLD, which accounts for 10% of the whole venom is a major cause of toxicity and lethality in humans [8,19]. This enzyme has high structural homology with PLD of the brown spider *Loxosceles intermedia*, and the cyclic PLD products directly contribute to the pathology of envenomation in humans [29]. Borchani et al. used a purified heminecrolysin (HNC); a 33 kDa protein from *H. lepturus* which has an identical lysophospholipase D activity as PLD which triggers dermonecrosis in rabbit skin [17]. Appel et al. showed that the recombinant isoforms of dermonecrotic toxins from *L. intermedia* show high dermonecrotic activity like *H. lepturus* venom [30]. Based on the sequence of rPLD1 of *H. lepturus* which has been previously determined by Torabi et al. with the gene accession number KY287766, the mutant isoform (His12Ala – His48Ala) was constructed and cloned into pET-22b (+) as mut-rPLD1 followed by expressing and purifying it as an expected 32 kDa protein [19]. Comparisons between the rPLD1 and mut-rPLD1 structures according to fluorescence spectroscopy, circular dichroism and output data obtained from the I-TASSER server [31] indicate that both proteins have high structural homology in both secondary and tertiary structures and it can be concluded the mut-rPLD1 can act as a suitable candidate to trigger immunity and produce antibodies against rPLD1 and crude venom of *H. lepturus*. Vuitika et al. demonstrated that mutation of His12 and His47 to Ala in PLD from *L. intermedia* causes complete inactivation of the enzyme without altering the enzyme structure [32]. Safari et al. developed a new rPLD1 toxoid by reacting rPLD1 with formalin for detoxification of rPLD1 through modification of histidine residues in the enzyme which resulted in formation uncommon imidazole derivatives and subsequently inactivation of the enzyme [33]. Borchani et al. revealed that inflammation and dermonecrosis in rabbit skin was inhibited when heminecrolysin was mixed with anti-Hl sera [18]. In the present study by mutation of His12 and His48 to Ala in rPLD1 of *H. lepturus*, a novel recombinant PLD was developed for immunization of the mice. This experiment is the first study that assesses a genetically modified single protein of the whole crude venom which possess a complete immunity in mice via production of antibody against the lethality of crude venom of *H. lepturus*. No previous similar experiments have been performed on scorpion venom. *H. lepturus*-rPLD1 is major toxin with extreme high enzyme activity that is able to hydrolyze the TNPAL-SM substrate in sphingomyelinase assay as its activity is 1.4-fold higher than the crude venom [19]. Similarly, LiRecDT1 and LiRecDT4 from *L. intermedia* are more active than the crude venom of *L. intermedia* [21]. Kalapothakis et al. illustrated this variety in sphingomyelinase activity of some PLD isoforms derived from *L. intermedia* [34]. Our experiment showed that mut-rPLD1 from *H. lepturus* at 10 and 15 µg was not able to hydrolyze any dosage of TNPAL-SM. Moreover, mut-rPLD1 had no dermonecrotic effect on rabbit skin, thus proving site directed mutagenesis of His12 and His48 to Ala completely inactivated the enzyme, corroborating previous studies in which mutation of these critical catalytic residues resulted in enzyme inactivation. Barry et al. performed Ouchterlony immunodiffusion tests for detection toxins of *Clostridium botulinum* against mice antisera [35]. Here, by using this technique we established that IgG molecules which are produced in the vaccinated mice were completely reactive to all three antigens; rPLD1, mut-rPLD1 and crude venom and the formed precipitin lines without spur indicates that all antigens have fully identical epitopes. Torabi et al. demonstrated by western blotting that horse antisera were immunoreactive against *H. lepturus*-PLD [19]. In our developed western blotting assay, the mice antisera were completely reactive to rPLD1, mut-rPLD1 and *H. lepturus*-PLD, corroborating that formed IgGs can bind to rPLD1 and *H. lepturus*-PLD, subsequently neutralizing their lethality. This pattern is similar with the test performed for antisera obtained from heminecrolysin in rabbits [18]. Torabi et al. demonstrated that injection of 1 µg of rPLD1 into rabbit skin causes inflammation, erythema and necrosis with area of about 0.7 cm^2^ in skin [19]. However, we showed that injection of higher amounts (2.5, 5, 20 and 40 µg) of mut-rPLD1 caused no necrotic lesions in rabbit skin, illustrating the non-toxic nature of this mutant protein. Torabi et al. demonstrated that intraperitoneal injection of one LD_100_ of rPLD1 equal to 3.7 µg causes death in all tested mice [19]. Conversely, after injecting higher doses (5, 10 and 30 LD_100_) of mut-rPLD1 in tested mice, all of them survived for up to 2 weeks after injection, confirming that mut-rPLD1 has no lethal activity. In order to evaluate the potency of this mutant recombinant protein as a vaccine candidate and its effectiveness in neutralizing the lethality of crude venom of *H. lepturus* scorpion, the following experiments were performed. The groups of mice vaccinated by mut-rPLD1 using Freund or alum adjuvants, were challenged by whole venom of *H. lepturus*. Their survival rate was documented for 2 weeks post-injection and over longer time periods. This survival strongly affirms that all mice were completely protected against wide range of different lethal dosages of crude venom (10 LD_100_, equal to 50 mg/Kg), thus illustrating a successful and 100% immunization in the mice. Heidarpour et al. determined the LD_50_ amount of *H. lepturus* crude venom as 5 mg/Kg in BALB/c mice [36]. These data are greatly in accordance with ELISA in which a raised antibody titer was observed at the first injection of mut-rPLD1 followed by reaching the plateau state from the 3rd injection to the 6th injection. The antisera were completely able to neutralize crude venom. Stimulation indexes in splenocytes proliferation assay in Freund-vaccinated and alum-vaccinated mice comparing to crude venom-treated groups are 3.54 and 3.25, respectively. The result was higher than the quantity due to PHA, which in turn indicates the successful immunization and extreme amounts of memory B cells in vaccinated mice at any time to encounter crude venom of *H. lepturus*. Valavi et al. observed slow manifestation of pathological effects due to toxicity of *H. lepturus*-venom in envenomed patients [13]. In that respect a new medical tool such as developed vaccine in the present study can perform worthy role in preventing the aforementioned effects in humans. The only strategy for scorpionism currently is serotherapy in which the crude venom is injected into the horses, followed by isolation of its serum and purification of antibodies against whole venom in a Fab fragments therapy manner [37], but this type of immunotherapy has very critical limitations in terms of specificity [38]. Since the *H. lepturus*-PLD is mainly responsible for toxicity and lethality in humans, this developed recombinant vaccine candidate can play a very effective role in preventing complications caused by *H. lepturus* scorpion envenomation. While serotherapy, which is often associated with severe pain, is a therapeutic strategy, conversely, vaccine injection is a preventive strategy in which the people experience minimum pain during the vaccination, while becoming highly resistant against *H. lepturus* envenomation.

## 4. Materials and Methods

### 4.1. Protein Structure Prediction

With the purpose of studying three-dimensional and two-dimensional structure of rPLD1 and mut-rPLD1, the amino acid sequences of both of them were documented on the I-TASSER Server (Iterative Threading ASSEmbly Refinement) available from Michigan University (Ann Arbor, MI, USA): http://zhanglab.ccmb.med.umich.edu/I-TASSER, by using the method of Yang et al. [31]. From the output data, the two models with the highest C-score (Confidence score) and the lowest RMSD were selected.

### 4.2. Circular Dichroism Spectroscopy (CD)

To determine the differences between the characteristics of secondary structures of rPLD1 and mutant-rPLD1, the far-UV (190–250 nm) CD spectrum analysis was performed. By this technique, fractions of both proteins that are in the alpha-helix conformation, the beta-sheet conformation and other possible conformations can be revealed. Both enzymes were dialyzed against 1X PBS (137 mM NaCl, 2.7 mM KCl, 4.3 mM Na_2_HPO_4_, 1.4 mM KH_2_PO_4_) at 4 °C to get a final concentration of 0.2 mg/mL. Their spectra were recorded using a 1 mm cuvette by a Jasco J-715 spectropolarimeter (Jasco Corporation, Tokyo, Japan). All tests were performed at a rate of 100 nm/min and using a response time of 1 s and 1 nm bandwidth. In this test the temperature of cuvettes was maintained constant at 25 °C.

### 4.3. Fluorescence Spectroscopy

For analyzing protein folding and three-dimensional structure studying, the steady-state fluorescence spectra of both rPLD1 and mut-rPLD1 were carried out with Perkin-Elmer LS 50B luminescence spectrometer (Perkin Elmer, Mundelein, IL, USA) in a 1.0 cm length quartz cell. The concentration of both proteins was adjusted to 50 µg/mL in phosphate buffer at pH 7.4 and the measurements were done at 25 °C. The excitation wavelength for monitoring intrinsic (tryptophan) fluorescence was 280 nm and excitation slit was 10 nm. The spectra then were collected ranging from 300 to 400 nm with emission slit of 5 nm

### 4.4. Cloning and Expression of Mut-rPLD1

The mutant gene construct of rPLD1 (His12Ala‒His48Ala) with a 6 × His-Tag at the C-Terminal flanked by *Nde*I and *Xho*I was synthetized by Biomatik Corporation (Cambridge, ON, Canada) and cloned into pET-22b (+) expression vector. This construct was then transformed into *E. coli* BL21 (DE3) bacterial cells as outlined below [39]. The lyophilized recombinant plasmid was dissolved in sterile TE buffer (100 ng/µL) and diluted at 1:10 with this buffer to get final concentration of 10 ng/ µL. The amount of 2 µLof this solution was then added to 100 µL of BL21 bacterial competent cells media in a microfuge tube, mixed gently and placed on the ice for 30 min, followed by heat shocking the mixture by placing it in water bath for 1 min at 42 °C. After taking the tube out of the water bath it was placed on the ice for 5 min more, and immediately 1 mL of Luria-Bertani broth (LB) was added to the tube. The tube was placed in a shaking incubator at 37 °C for 1 h at 220 rpm so that the bacterial cells can recover. The tube was centrifuged at 3600× *g* for 5 min at room temperature so bacterial cells were precipitated. The supernatant was discarded and bacterial pellet was plated on LB agar plate (with 100 µg/mL concentration of ampicillin) and incubated at 37 °C for an overnight. A well-separated single colony of the transformants was selected and inoculated into 10 mL of autoclaved LB broth (containing 100 µg/mL of ampicillin) and incubated at 37 °C for an overnight at 220 rpm. From this overnight culture, 1 mL was then sub-cultured in 100 mL of fresh and autoclaved LB broth containing 100 µg/mL of ampicillin and incubated at 37 °C in shaker incubator at 180 rpm until the optical density (O.D) at 600 nm wavelength reached 0.6. For induction, isopropyl-β-D-thiogalactoside (IPTG, Thermo Fisher Scientific Co, Waltham, MA, USA) at a final concentration of 0.1 mM was added into the culture and the gene was expressed at 30 °C for 3.5 h in shaker incubator with vigorous shaking. The cells were harvested by centrifugation (10,000 g, 7 min, 4 °C) (Sigma 3–18 K, Osterode am Harz, Germany) and the resultant pellet was frozen at −20 °C for an overnight. All of these experiments were performed in parallel for rPLD1 as well.

### 4.5. Purification of Mut-rPLD1

Bacterial pellet was suspended in 2 mL of lysis buffer (300 mM NaCl, 50 mM NaH_2_PO_4_ and 10 mM Imidazole; pH 8), then placed in dry ice for 30 min and thawed and disrupted through sonication (Hielscher Co, Teltow, Germany) with short pulses (20 pulses of 20 s with a 40 s pause between them at 80% amplitude). The cell lysate was subjected to centrifugation at 10,000 g at 4 °C for 10 min. The pellet was discarded and bacterial supernatant was separated by affinity chromatography against His‒Tag. The Ni-NTA agarose (Qiagen Co, Hilden, Germany) column was washed and equilibrated with washing buffer (300 mM NaCl, 50 mM NaH_2_PO_4_ and 20 mM Imidazole; pH 8). The supernatant containing soluble protein was incubated with 1 mL of Ni^2+^‒NTA agarose beads at 4 °C for 1 h. This suspension was then loaded onto the column and the packed gel was washed by washing buffer (as mentioned above) to remove any impurities. The pure mutant-rPLD1 was obtained with elution buffer (300 mM NaCl, 50 mM NaH_2_PO_4_ and 250 mM imidazole; pH 8). The eluted fractions were then collected, pooled and dialyzed against 1X PBS buffer. For checking the true purity of the eluted mutant-rPLD1, the final fraction was analyzed by SDS-PAGE technique. Finally, the protein concentration was determined using BCA Protein Assay Kit according to manufacturer instructions (iNtRON Biotechnology Co, Seoul, South Korea). All of this process was also done simultaneously for rPLD1.

### 4.6. SDS-PAGE Analysis for Assessing Proteins

For separating rPLD1 and mutant-rPLD1, the sodium dodecyl sulfate-polyacrylamide gel electrophoresis (SDS-PAGE) technique was applied according to Laemmli et al. [40]. For preparation of protein samples, rPLD1, mutant-rPLD1 and bacterial lysate supernatant were separately mixed with sample buffer, 15–20 µg of each sample subjected to SDS-PAGE. All samples were then heated at 95 °C for 5 min. Thereafter, each sample was pipetted into one of the wells in the gel. Also protein molecular weight marker (Thermo Fisher Scientific Co. Waltham, MA, USA.) was loaded into its own well in the gel. Then, a 15-mA electrophoretic flow was applied to the gel for initial 30 min, followed by 25-mA for 2 h in order to migrating of negatively charged proteins through the gel in the direction of anode pole. Finally, the gel was brought out of the glass plates and placed in the Coomassie Brilliant Blue^®^ R-250 (Thermo Fisher Scientific Co. Waltham, MA, USA.) solution for staining for 12 h at room temperature followed by immersing gel into the destaining solution until background of the gel was fully destained and protein bands became visible.

### 4.7. In Vitro Sphingomyelin Hydrolysis Activity Assay of Mut-rPLD1

Sphingomyelinase activity of the mut-rPLD1 was assessed by measuring the enzyme-catalyzed hydrolysis of a SM synthetic colored derivative, Trinitrophenyl-aminolauryl-shpingomyelin (TNPAL-SM) (Sigma Chemical Co. St Louis, MO, USA) according to the previous study [17]. Increased amounts of mut-rPLD1, including 10 and 15 µg were incubated with 190 µL of incubation buffer containing 250 mM Tris-HCl, 20 mM MgCl_2_, 0.1% Triton X-100 and 60 nM of TNPAL-SM at pH 7.4. The reaction mixtures were slowly shaken at 37 °C for 2 h. Subsequently the reactions were stopped by adding 375 µl of isopropanol/heptane/H_2_SO_4_ (40:10:1 v/v). Then 200 µL of both heptane and water added to each reaction. The tubes were centrifuged at 660 g for 5 min to separate the two phases. The upper phase was transferred to a 96-well microplate and the optical density was read at 410 nm. The rPLD1 was used as the positive control and the sphingomyelin hydrolysis was expressed as the number of nmol of TNPAL-SM hydrolyzed per mg of mut-rPLD1 (1 nmol of hydrolyzed TNPAL-SM corresponds to 0.023 absorbance units).

### 4.8. Non-Lethality Test of Mut-rPLD1 in the Mice

It has been shown that the rPLD1 from *H. lepturus* causes death in mice with LD_100_ = 3.7 µg according to a previous study [19]. The mortality caused by rPLD1 in the mice was recorded during 4 days after intraperitoneal (IP) injection. Four groups of five mice were chosen for intraperitoneal injection by different doses of mut-rPLD1. The first group received 3.7 µg (equivalent of 1 LD_100_), the second group 18.5 µg (= 5 LD_100_), the third group 37 µg (= 10 LD_100_) and the fourth group 111 µg (= 30 LD_100_). All the mut-rPLD1 injections were prepared in 100 µl of sterile PBS. Another group of five mice received 3.7 µg of rPLD1 (= 1 LD_100_) as negative control. All the groups were studied and monitored for 2 weeks.

### 4.9. Non-Dermonecrotic Test of Mut-rPLD1 on Rabbit Skin

As previously demonstrated, 1µg of rPLD1 triggers dermonecrotic lesion of about 0.7 cm^2^ in rabbit skin [19]. In order to determine whether mut-rPLD1 is capable of inducing dermonecrotic lesions, four different doses (2.5, 5, 20 and 40 µg) of this toxoid were intradermally injected into the rabbit skin. As positive controls, rabbit was injected with the same doses of rPLD1 and crude venom respectively. PBS was used as negative control and the dermonecrotic effects of all injected toxins and toxoids were checked at 24 h post-injection.

### 4.10. Immunization of the Mice by Mut-rPLD1

Eight-weeks-old male and healthy BALB/c mice (20–22 gr) were purchased from the Pasteur Institute of Iran (Tehran, Iran) and kept in 12:12 h light/dark cycle at the room temperature of 22 ± 1 °C and humidity of 50 ± 5% and were given a standard food regimen and water. All of the animal trials in this topic were executed according to the recommendations of the school’s ethics committee and animal care and use agreement at Pasteur Institute of Iran (approval number: IR.PII.REC.1394.38). They were assigned into two 60 mice groups. The first group was chosen for vaccinating with toxoid-Freund’s adjuvant and the second group for toxoid-alum adjuvant as described in detail below. The aim of using both Freund’s adjuvant and alum adjuvant is comparison between their final immunogenic effect on the immune system of the mice. At the first step of all of these experiments the pre-immune sera from non-immunized mice were collected and kept at −20 °C until the usage.

#### 4.10.1. Immunization by Toxoid-Freund’s Adjuvant

Sixty mice received an initial subcutaneous injection of 10 µg of mutant enzyme (toxoid). This amount of toxoid was increased to 100 µl with sterile PBS and then mixed completely with 100 µl of Complete Freund’s Adjuvant (CFA, Sigma Co. Roedermark, Germany) and homogenized by stirring for 4 h and incubated for 24 h at 4 °C, so a homogeneous solution of toxoid and CFA was obtained. The subsequent injections were also carried out subcutaneously on days 14, 28, 42, 56 and 70 with the same condition but with Incomplete Freund’s Adjuvant (IFA, Sigma Co. Roedermark, Germany). Before each injection the blood samples were taken from the mice and their serum was separated and kept at ‒20 °C until use. These processes of blood sampling were performed consecutive at the end of the weeks 2, 4, 6, 8 and 10. Also six mice were received only sterile PBS-Freund’s adjuvant under the same conditions as negative controls.

#### 4.10.2. Immunization by Toxoid-Alum Adjuvant

In this series of experiments each of 60 other mice received 10 µg of toxoid. This amount of toxoid was mixed with 100 µL of sterile PBS and then mixed with 100 µl of Alum adjuvant (Thermo Fisher Scientific Co. Waltham, MA, USA.) followed by stirring until a homogenous solution was obtained. 200 µL of this solution was subcutaneously injected to each mouse on days 1, 14, 28, 42, 56 and 70. In this series of experiments, as in the Freund’ adjuvant experiments, the blood samples were taken from the mice before each injection and their sera were separated and kept at −20 °C until use. Here also, 6 mice that only received sterile PBS-alum under the same conditions were considered as negative controls. The purpose of these series of experiments was to compare the efficacy between Freund’s adjuvant and alum adjuvant in immunization process of the mice.

### 4.11. Indirect ELISA for Detection of Raised Antibody in Immunized Mice

The anti-toxoid in the mice sera was measured by an Indirect ELISA method as outlined below. A 100 µL solution of rPLD1 (10 µg/mL) was mixed with 100 µL of coating buffer (0.1 M Carbonate-Bicarbonate buffer in deionized water, pH adjusted to 9.6) and dispensed into individual wells of a 96-Well flat bottom microtiter plate (Nunc, Sigma Co. St. Louis, MO, USA). The plate was covered with an adhesive plastic and incubated at 4 °C for an overnight. The coating buffer was removed and all wells were washed 3 times with 300 µL of phosphate-buffered saline (PBS, 1×). The solutions were removed by flicking the plate over a sink and the remaining drops were removed by patting the plate on a paper towel. All the wells were filled by adding 100 µl of blocking buffer, 2% bovine serum albumin (BSA, Merck Co. Darmstadt, Germany) in PBS for blocking the remaining protein-binding sites in the coated wells and incubated at room temperature for 1 h with gentle shaking. For removing the unbound BSA molecules from the wells, the plate was patted slightly for several times on a paper towel. 100 µL of the sera from immunized mice (mice immunized by toxoid-Freund’s adjuvant) were diluted with 1 × PBS in serial dilution from 1:500 to 1:64,000, then adding each dilution to one well and the plate was incubated at 37 °C for 1 h with gentle shaking. After six times of washing each well with 300 µl of PBST (PBS-0.05% Tween 20), 100 µL of goat anti-mouse IgG antibody labelled with horseradish peroxidase (HRP) (Sigma Co. St. Louis, MO, USA) diluted with PBS at 1:3000 titer was added to each well and incubated at 37 °C for 1 h. By dispensing of 100 µl of peroxidase substrate, 3,3ʹ,5,5ʹ-Tetramethylbenzidine (TMB, Pishtaz Teb Zaman Diagnostics; Tehran, Iran) to each well and incubating the plate at 37 °C in dark for 15 min, the TMB reaction after developing sufficient color was stopped by adding each well 100 µl of stopping solution (2N H_2_SO_4_). The absorption of each well was measured at 450 nm with a plate reader (EPOCH, BioTek Instruments, Winooski, VT, USA). All the experiments were performed in triplicate.

This ELISA assay was carried out in six series according to which of the antigens (rPLD1, mut-rPLD1 or crude venom) has been coated inside the wells and also based on the sera received either from the mice immunized with Freund’s adjuvant or alum adjuvant as following: anti-toxoid sera from mice immunized with toxoid-Freund’s adjuvant in 1st run against rPLD1 (as explained above), in 2nd run against mut-rPLD1, 3rd run against crude venom; and so in regard to the antisera from mice immunized with toxoid-Alum adjuvant in 4th run against rPLD1, in 5th run against mut-rPLD1 and in 6th run against crude venom was performed.

### 4.12. Double-Immunodiffusion Assay (Ouchterlony Test)

For analyzing the antigens and antibodies this immunological technique was applied [41]. Briefly, 1 g of agarose powder was dissolved in 100 mL of sterile 1× PBS in a glass beaker (final concentration of 1%) followed by heating at 100 °C for 10 min to achieve a homogeneous solution. 0.5 mg of sodium azide was then added as antimicrobial agent to prevent unwanted bacterial growth. The solution was slowly poured onto the plastic plate on a horizontal surface and waited 30 min for solution to solidify and form flat gel. The gel was then punched with disposable vacuum plastic pipette to form a series of apart holes in it. 50 µL of mice anti-toxoid sera was placed in the middle hole, 50 µg of rPLD1 in the adjacent hole, 50 µg of mutant-rPLD1 in the other adjacent hole, the next one 500 µg of crude venom (PLD consists 10% of the whole crude venom) and the last one was filled with 100 µL of PBS solution as negative control. After antisera and other antigens were absorbed into the gel during the initial 15 min, all the holes refilled three times with the respective antisera and antigen solutions for obtaining more precise result. The plate was then covered with its lid and kept in humidified chamber (a box with wet cotton) on a horizontal surface at 37 °C for 24 h. This test was performed for both antisera of mice immunized by Freund’s adjuvant and alum adjuvant.

### 4.13. Western Blotting Assay

The rPLD1, mut-rPLD1 and crude venom were subjected to 15% SDS-PAGE in reducing sample buffer. PVDF membrane (Sigma Co, Darmstadt, Germany) was incubated in transfer buffer (25 mM trisaminomethane, 190 mM gGlycine and 20% methanol in 1× PBS) for 5 min. After electrophoresis was completed, the proteins were then electroblotted into the prepared PVDF membrane by semi-dry blotting system (Bio-Rad Co. Hercules, CA, USA) under 18-volt current for 30 min to stabilize the proteins on the membrane. The PVDF membrane was floated inside the 4% Skim milk in 1X PBS buffer at 4 °C for an overnight with mild shaking to block the remaining binding sites. Then incubated in the mice anti-sera (diluted at 1:1000 titer) for 1 h at room temperature as primary antibody. The membrane was washed 3 times for 5 min with PBST (PBS-0.05% Tween 20) and incubated with Goat anti-mouse IgG antibody labelled with HRP (diluted at 1:3000 titer) at room temperature for 1 h. After washing the membrane 6 times with PBST, the rPLD1, mut-rPLD1 and crude venom were developed by submerging membrane into the peroxidase substrate solution; DAB (3,3ʹ-diaminobenzidine; Thermo Fisher Scientific Co. Waltham, MA, USA) according to the manufacturer’s instruction.

### 4.14. Splenocytes Proliferation Assay

The mice which have been vaccinated by mut-rPLD1-Freund’s adjuvant were sacrificed by dislocation of their spinal cord 2 weeks after the last injection and their spleens were dissected out under aseptic conditions. All spleens were washed by physiological saline solution and then homogenized in a sterile Petri dish containing 10 mL of RPMI 1640 with 10% FBS by gentle plunging them against the bottom of the dish with a syringe plunger. The resultant suspension was sieved through a 70 µm nylon cell strainer into a sterile 15 mL conical tube to avoid tissue debris. The liquid leaking out of the strainer which now contains splenocytes and RBCs was layered over Ficoll density gradient solution, centrifuged at 400× *g* for 40 min. The middle layer which contains isolated splenocytes was aspirated with a Pasteur pipette and washed with 5 mL of sterile RPMI 1640 and centrifuged at 400× *g* for 10 min. The supernatant was discarded and the precipitated splenocytes were resuspended in 5 mL of RPMI 1640–10% FBS and centrifuged at 400× *g* for 10 min. This procedure was repeated twice. The cell pellet was resuspended in 5 mL of RPMI-10% FBS and cell counting was performed by hemocytometer under a light microscope. Finally, the cell count was adjusted to 2 × 10^5^ cells per 100 µL of RPMI-10% FBS (supplemented with 1% penicillin/streptomycin). The MTT assay was performed to investigate the splenocyte proliferation. Each of 5 wells of a 96-well sterile cell culture plate was filled with 100 µl of this suspension. Then the cells were cultured in RPMI 1640–10% FBS and were incubated for 24 h at 37 °C with 5% of CO_2_ and 90% humidity. 20 µg of mut-rPLD1 was added to the first well, 20 µg of rPLD1 to the next well and 200 µg of crude venom to the next one (PLD consists 10% of the whole venom). As a positive control 100 µL of phytohemagglutinin M (10 µg/mL; PHA-M; Sigma Co. St. Louis, MO, USA.) was added to the next well and the last well which only contains untreated cells, served as negative control. The plate was reincubated for 24 h at 37 °C with 5% of CO_2_ and 90% humidity. 20 µL of 5 mg/mL 3-(4,5-dimethyl-2-thiazolyl)-2,5-diphenyl-2*H*-tetrazolium bromide solution (MTT; Sigma Co. St. Louis, MO, USA.) containing in PBS was added to all wells and plate was incubated for 4 h more followed by adding 100 µl of DMSO (dimethyl sulfoxide; Merck Co. Darmstadt, Germany) to each well and waited for 15 min. The absorption of each well was measured at 570 nm using plate reader (EPOCH). The stimulation index “S.I” was expressed as average MTT OD values in treated groups divided by average MMT OD values in non-treated groups. All these experiments were carried out in triplicate. This assay of splenocytes proliferation was also done for alum-vaccinated mice.

### 4.15. Challenging Tests in Immunized Mice

The mice immunized by toxoid were challenged one week after the last injection. In the case of the mice which have been immunized by toxoid-Freund’s adjuvant, the first group of them was divided into five groups of six mice. Each mouse in group 1 received 10 (37 µg), group 2 = 20 (74 µg), group 3 = 50 (185 µg), group 4 = 100 (370 µg) and the group 5 = 200 (740 µg) LD_100_ of the rPLD1. Also the other 30 mice from these mice were assigned to five groups of six mice and received different doses of crude venom, as group 1 received 1 (100 µg), group 2 = 2 (200 µg), group 3 = 5 (500 µg), group 4 = 7 (700 µg) and the group 5 = 10 (1000 µg) LD_100_ of the crude venom.

The other 60 mice that were immunized by toxoid-alum adjuvant received the same doses of rPLD1 and crude venom respectively as mentioned above. All the injections were done intraperitoneally and mice were monitored for 2 weeks. Those that survived were regarded as the survivors and non-immunized mice were regarded as the negative controls.

### 4.16. Statistical Analysis

Statistical analyses were performed with GraphPad Prism V.8.0 software (GraphPad Software Inc, San Diego, CA, USA) The *p* value < 0.001 was calculated by using ANOVA test for significant changes. Tukey’s multiple comparisons test was used as post hoc test. The results are presented as mean ± standard deviation (SD).

## 5. Conclusion

In conclusion, mut-rPLD1 can significantly stimulate antibody production in mice and thus neutralize the toxic and lethal effects of whole venom. According to the obtained results, this toxoid can perform as a valuable tool in order to trigger active immunization against *H. lepturus* scorpion stings. This protein as a modified recombinant protein and as an immunogen can produce antibodies and through more biotechnological procedures will play a role as an effective vaccine candidate instead of serotherapy in the future for humans.

## Figures and Tables

**Table 1:** In vivo non-lethality test of mut-rPLD1 in mice. All mice that received different doses of mut-rPLD1 were survived after 2 weeks post-injection. While negative control mice that received 3.7 µg of rPLD1 were dead during 12 h post-injection.

Groups	Number of Mice	Mut-rPLD1 (µg)	Survival Rate
**1**	5	3.7	5/5
**2**	5	18.5	5/5
**3**	5	37	5/5
**4**	5	111	5/5
		**rPLD1 (µg)**	
**Negative control**	5	3.7	0/5

**Table 2:** In vivo vaccination trials in immunized mice. (**a**): One LD_100_ of rPLD1 is equal to 3.7 µg. (**b**): One LD_100_ of *H. lepturus* scorpion crude venom is equal to 100 μg (**c**): The mice that only received Adjuvant + PBS (negative controls).

Mice Vaccinated by Mut-rPLD1-Freund/Alum Adjuvant							
Number of Mice		rPLD1 Challenge (LD_100_s)^(a)^	Survival Rate	Number of Mice		Crude Venom Challenge (LD_100_s)^(b)^	Survival Rate
6		10	6/6	6		1	6/6
6		20	6/6	6		2	6/6
6		50	6/6	6		5	6/6
6		100	6/6	6		7	6/6
6		200	6/6	6		10	6/6
**Neg cont** ^**(c)**^		1	0/6	**Neg cont** ^**(c)**^		1	0/6
	6				6		
